# Association between the weight-adjusted waist index and the odds of type 2 diabetes mellitus in United States adults: a cross-sectional study

**DOI:** 10.3389/fendo.2023.1325454

**Published:** 2024-01-16

**Authors:** Dongdong Zheng, Suzhen Zhao, Dan Luo, Feng Lu, Zhishen Ruan, Xiaokang Dong, Wenjing Chen

**Affiliations:** ^1^ Shandong University of Traditional Chinese Medicine, Jinan, Shandong, China; ^2^ Dongying People’s Hospital (Dongying Hospital of Shandong Provincial Hospital Group), Dongying, Shandong, China; ^3^ Affiliated Hospital of Shandong University of Traditional Chinese Medicine, Jinan, Shandong, China

**Keywords:** weight-adjusted waist index, type 2 diabetes mellitus, NHANES, United States adults, cross-sectional study

## Abstract

**Objective:**

To examine the association between the weight-adjusted waist index (WWI) and the odds of type 2 diabetes mellitus(T2DM)among U.S. adults.

**Methods:**

Data from the National Health and Nutrition Examination Survey (NHANES) spanning six years (2007–2018) were utilized, encompassing 31001 eligible participants. Weighted multivariate logistic regression models and smoothed fit curves were employed to assess the association between WWI and the odds of T2DM, as well as dose-response relationships in the overall population and the odds of T2DM in various subgroups.

**Results:**

In the fully adjusted continuous model, each one-unit increase in WWI was associated with a 1.14-fold increase in the odds of T2DM within the entire study population (2.14 [1.98,2.31], P < 0.0001). In the fully adjusted categorical model, when using the lowest tertile of WWI (T1) as the reference group, the second tertile (T2) and the third tertile (T3) were associated with a 0.88-fold (1.88 [1.64,2.17], P < 0.0001) and a 2.63-fold (3.63 [3.11,4.23], P < 0.0001) increase in the odds of T2DM. These findings indicated a positive correlation between WWI values and the odds of T2DM, aligning with the results of the smoothed-fitted curves. In the analysis of subgroups, in addition to maintaining consistency with the overall population results, we found interactions between age and hypertension subgroups.

**Conclusion:**

In conclusion, WWI was found to be positively associated with the odds of T2DM in U.S. adults.

## 1 Introduction

Type 2 diabetes mellitus (T2DM) stands as a chronic metabolic disorder known for its hallmark features of insulin resistance and elevated blood glucose levels. Once diagnosed, it typically proves challenging to reverse, and it frequently leads to complications affecting the kidneys, retinal health, cardiovascular system, neural function, and liver. Regrettably, effective treatments for this condition have been lacking (1). Recent statistics indicate a concerning trend, with the prevalence of diabetes projected to surge to 578 million individuals by 2030 (2). Therefore, we must take effective measures to identify people at risk of developing diabetes and to give relevant preventive guidance and advice promptly, to achieve the goal of slowing down the onset and development of diabetes.

Globally, the rates of obesity have reached epidemiological levels and are impacting an increasing population of individuals (3–5). Studies have demonstrated a strong association between obesity and the development of type 2 diabetes (6). Although Body Mass Index (BMI) is the most commonly used method to assess obesity, an important limitation of applying BMI is that it does not reflect the true body fat distribution (7). Waist circumference (WC) is a simple and reliable method for assessing abdominal obesity and has been used to measure total body fat (8), but fails to distinguish between subcutaneous and visceral fat (9). It has been shown that visceral fat produces more free fatty acids than subcutaneous fat, which increases insulin resistance and the risk of diabetes (10, 11). Quantifying visceral fat and muscle mass by applying Computer Tomography (CT) or Magnetic Resonance Imaging (MRI) lacks feasibility in clinical work. Therefore, Park Y et al. proposed a novel index of obesity called the “weight-adjusted waist circumference index (WWI)” (12). The WWI is calculated as waist circumference (WC) divided by the square root of body weight, which not only weakens the relationship with BMI but also combines the advantages of WC to provide a better reflection of body fat distribution and muscle mass, as well as reflecting the problem of central obesity, which is not related to body weight (13). Therefore, WWI has potential advantages as a predictor of obesity.

Previous studies have shown a positive association between WWI and the prevalence of type 2 diabetes (14, 15), but these studies have been conducted in Asian countries, and there is a lack of research on the association between WWI and the prevalence of type 2 diabetes in the US population. Therefore, we applied a large sample of data obtained from the NHANES database from 2007 to 2018 to further validate the potential relationship between WWI and the odds of T2DM.

## 2 Methods

### 2.1 Study population

NHANES, a nationally representative cross-sectional study, was purposefully designed to evaluate the health and nutritional status of an ambulatory population within the United States. The U.S. Centers for Disease Control and Prevention secured approval from the Research Ethics Review Board for this study, and all participants provided written informed consent, obviating the need for further ethical review. The present study adhered to the guidelines outlined in the Epidemiologic Statement for Enhanced Reporting of Observational Studies (16).

**Figure 1:**
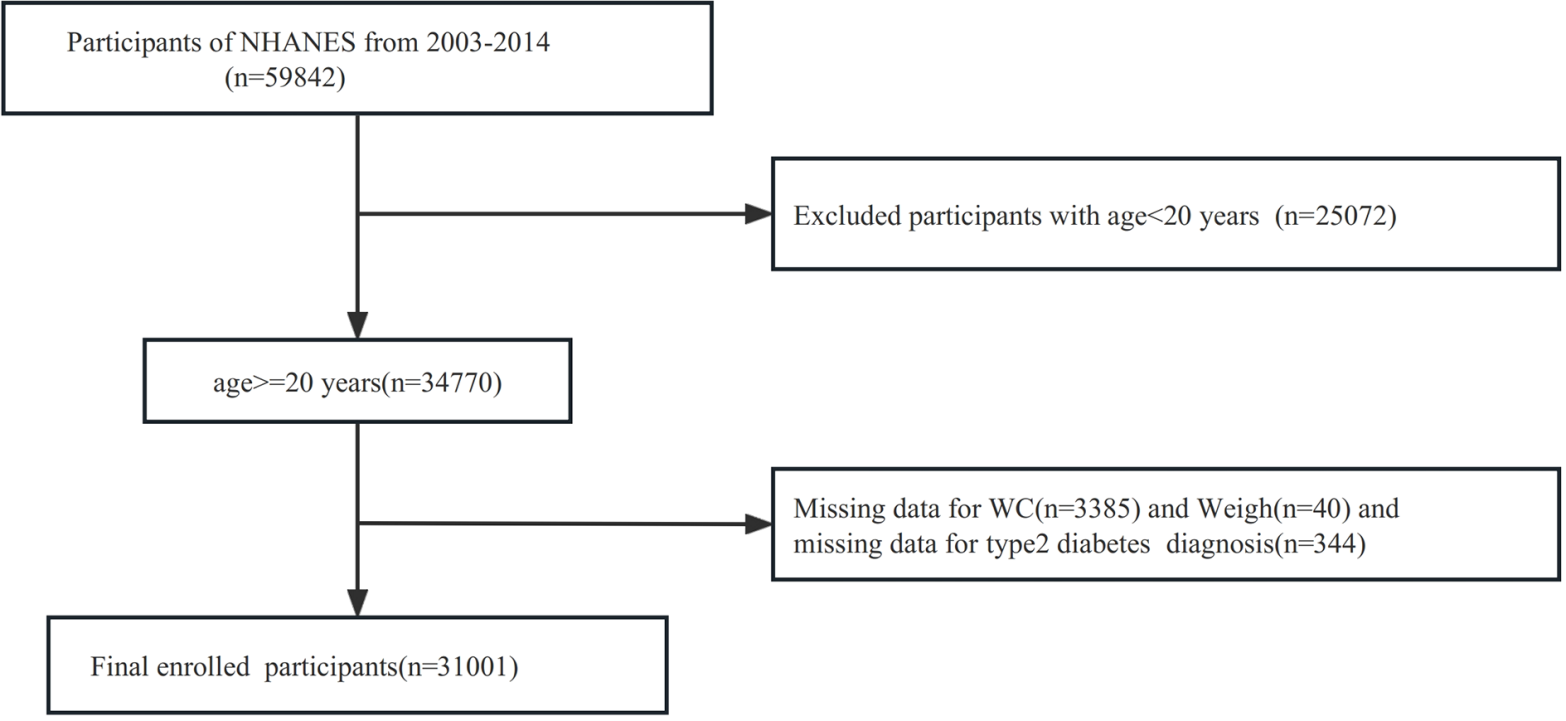
Flowchart of participants’ selection.

For the investigation, a comprehensive screening of the entire 2007-2018 NHANES database was carried out, encompassing a total of 59842 participants. To ensure the relevance and accuracy of the study, specific exclusion criteria were applied. Participants who were younger than 20 years old were excluded, resulting in the exclusion of 25072 participants. Additionally, participants with missing data on the waist circumference were excluded, leading to the exclusion of 3385 participants. Participants with missing data on weight were excluded, leading to the exclusion of 40 participants. Moreover, those with incomplete records regarding a diagnosis of T2DM were also excluded, accounting for an additional 344 participants. Consequently, the final dataset analyzed for the study consisted of 31001 subjects. Further details are illustrated in **Figure 1**.

### 2.2 Diagnosis of T2DM

Under the 2013 U.S. Diabetes Guidelines (17), T2DM was categorized as meeting any of the following criteria: 1) self-reported physician-diagnosed T2DM; 2)Currently taking hypoglycemic drugs or injecting insulin;3) random blood glucose level equal to or exceeding 11.1 mmol/L; 4) glycosylated hemoglobin (HbA1c) level equal to or exceeding 6.5%; 5) fasting blood glucose level equal to or exceeding 7.0 mmol/L; 6) 2-h blood glucose level (determined by oral glucose tolerance test (OGTT)) equal to or exceeding 11.1 mmol/L. In the analysis, the incidence of T2DM was regarded as the outcome of interest.

### 2.3 Calculation of WWI

WWI (cm/√kg) was measured by calculating WC (cm) divided by the square root of the weight (kg). Anthropometric measurements were recorded by trained medical personnel and specialized recorders to ensure the exact accuracy of the data. Body weight was determined using a digital scale. Participants were asked to wear their examination clothing, and then stand barefoot on the digital scale with arms held close to their bodies and gaze fixed straight ahead, as outlined previously (18). WC was calculated using a tape measure, which was positioned at the intersection of the midaxillary line and the horizontal line just above the outermost upper point of the right kneecap (19). The WWI value was used as an exposure variable in this study.

### 2.4 Covariates

Covariates, such as age, gender, race/ethnicity (Non-Hispanic white, Non-Hispanic black, Mexican-American, etc.), educational level (less than high school, high school or equivalent, college or above), marital status (married/living with partner, never married, widowed/divorced/separated), family poverty-to-income ratio (PIR), smoking status (never, now, former), alcohol user (never, former, mild, moderate, heavy), Physical activity(PA, Mets), Alanine aminotransferase (ALT, U/L), Aspartate aminotransferase (AST, U/L), creatinine(umol/L), uric acid(UA,umol/L), triglycerides (TG, mmol/L), high-density lipoprotein-cholesterol (HDL-C, mmol/L), total cholesterol (TC, mmol/L). Hypertension was defined as meeting any of the following criteria: 1) self-reported physician diagnosis of hypertension; 2) current use of antihypertensive medication; 3) an average of three measurements of systolic blood pressure (SBP) exceeding 140 mmHg and/or diastolic blood pressure (DBP) exceeding 90 mmHg. Hyperlipidemia can be diagnosed by meeting any of the following conditions: 1) Diagnosed as hypertriglyceridemia (TG>=150mg/dL or>=1.70mmol/L); 2) Diagnosis of hypercholesterolemia can be made by meeting any of the following conditions: 1. TC>=200mg/dL (>=5.18mmol/L) 2. Low-density lipoprotein-cholesterol(LDL-C)>=130mg/dL (>=3.37mmol/L) 3. Male HDL<40mg/dL (<1.04mmol/L), female HDL<50mg/dL (<1.30mmol/L); 3) Currently taking lipid-lowering medication. The diagnosis of coronary heart disease (CHD) was determined based on self-reported responses to the NHANES questionnaire. The calculation of physical activity is based on multiplying the number of days (in days/week) of specific activities per week by the duration of specific activities per day (in minutes/day) to calculate the resulting exercise metabolic equivalents (METs). Stroke diagnoses were derived from self-reported stroke diagnoses from the questionnaire. Some covariates had missing values, although the extent of missing data for each variable was less than 20%. These missing values were addressed through the utilization of the “mice” R package, employing multiple interpolations to impute the missing data for continuous variables.

### 2.5 Statistical analysis

The statistical analysis was conducted using R 4.3.1 software. The analysis incorporated the variables for weights, sdmvstra, and sdmvpsu. Continuous variables were expressed as mean ± standard deviation, while categorical variables were presented as numbers and percentages. To make the comparison between the two groups, weighted Student’s t-test, Mann-Whitney U test, and the Chi-square test were utilized.

To explore the relationship between WWI level and the odds of developing T2DM, the multivariate logistic regression model was employed. Odds ratios (ORs) and their corresponding 95% confidence intervals (CIs) were computed using the “survey” R package. Model 1 was unadjusted for any covariates. Model 2 was adjusted for age, gender, and race/ethnicity. Model 3 further included adjustments for educational level, marital status, PIR, smoking status, alcohol user, ALT, AST, creatinine, UA, TC, TG, HDL, hypertension, CHD, stroke, and Hyperlipidemia. The multivariate logistic regression model was divided into categorical and continuous models. In the categorical model, WWI values were categorized into thirds, and nonlinear trends were assessed by treating the median value of each trichotomy as a continuous variable.

In addition, subgroup analysis was conducted to indicate whether age, gender, race/ethnicity, hypertension, CHD, stroke, and Hyperlipidemia were associated with the odds of T2DM in different population subgroups. The level of statistical significance was set at P<0.05.

## 3 Results

### 3.1 Characteristics of the study population

A total of 31001 participants were included, and **Table 1** shows the demographic and clinical features of participants by tertiles of baseline WWI values. All variables were statistically significant among the three WWI subgroups. Compared with participants in the T1 group, participants in the T3 group were mostly female, older, non-Hispanic white, less educated, Divorced/widowed/separated, exhibited a higher prevalence of former smokers and previous drinkers, had lower PIR, creatinine, PA and HDL, had higher values of WC, Weight, ALT, AST, UA, TC, TG, and BMI. Besides, they had higher incidence rates of hypertension, CHD, Hyperlipidemia, T2DM, and stroke.

**Table 1:** Basic characteristics of participants by weight-adjusted-waist index tertile.

variable	total	T1(=<10.70)	T2(11.70-11.44)	T3(>11.44)	Pvalue
Age (years old)	47.40 ± 0.22	38.93 ± 0.27	49.03 ± 0.25	56.81 ± 0.28	< 0.0001
Sex, (%)					< 0.0001
Female	15719(50.7)	4206(42.33)	5030(49.81)	6483(63.96)	
Male	15282(49.3)	6125(57.67)	5301(50.19)	3856(36.04)	
Race/ethnicity, (%)					< 0.0001
Mexican American	4668(15.06)	970(5.98)	1709(9.84)	1989(10.50)	
Non-Hispanic Black	6612(21.33)	2765(13.40)	2106(10.09)	1741(9.32)	
Non-Hispanic White	12617(40.7)	4184(66.47)	4013(65.04)	4420(67.50)	
Others	7104(22.92)	2412(14.15)	2503(15.03)	2189(12.69)	
Educational level, (%)					< 0.0001
College or above	5128(16.54)	2145(22.55)	1736(17.86)	1247(12.04)	
High school or equivalent	18289(58.99)	6476(66.21)	6104(66.29)	5709(66.34)	
Less than high school	7584(24.46)	1710(11.23)	2491(15.85)	3383(21.63)	
Marital status, (%)					< 0.0001
Divorced/widowed/separated	6830(22.03)	1488(11.82)	2148(17.77)	3194(27.32)	
Married/living with partner	18439(59.48)	5805(60.28)	6687(68.69)	5947(60.98)	
Never married	5732(18.49)	3038(27.90)	1496(13.53)	1198(11.70)	
PIR	2.99 ± 0.03	3.16 ± 0.04	3.03 ± 0.04	2.70 ± 0.04	< 0.0001
Smoking status, (%)					< 0.0001
Former	7335(23.66)	1743(18.20)	2571(26.51)	3021(30.66)	
Never	17282(55.75)	6046(60.11)	5715(54.18)	5521(51.17)	
Now	6384(20.59)	2542(21.70)	2045(19.32)	1797(18.17)	
Alcohol user, (%)					< 0.0001
Former	4311(13.91)	883(6.83)	1440(12.11)	1988(16.74)	
Heavy	5694(18.37)	2399(23.99)	1904(19.25)	1391(14.69)	
Mild	9456(30.5)	3387(34.99)	3199(33.61)	2870(32.39)	
Moderate	4315(13.92)	1674(17.57)	1481(16.68)	1160(13.35)	
Never	7225(23.31)	1988(16.62)	2307(18.34)	2930(22.84)	
Waist circumference (cm)	99.26 ± 0.23	87.87 ± 0.18	100.60 ± 0.18	112.86 ± 0.26	< 0.0001
Weight (kg)	82.66 ± 0.24	75.86 ± 0.25	83.89 ± 0.29	90.28 ± 0.42	< 0.0001
Alt(U/L)	25.30 ± 0.16	23.57 ± 0.19	26.86 ± 0.34	25.79 ± 0.27	< 0.0001
Ast(U/L)	25.22 ± 0.12	24.64 ± 0.15	25.66 ± 0.23	25.48 ± 0.23	< 0.001
UA(umol/L)	322.38 ± 0.79	310.26 ± 1.17	324.03 ± 1.22	336.62 ± 1.13	< 0.0001
Creatinine(umol/L)	77.87 ± 0.23	78.59 ± 0.31	77.26 ± 0.35	77.63 ± 0.36	0.01
Total cholesterol (mmol/L)	5.01 ± 0.01	4.89 ± 0.02	5.11 ± 0.02	5.04 ± 0.02	< 0.0001
Triglyceride (mmol/L)	1.72 ± 0.02	1.41 ± 0.02	1.84 ± 0.02	2.00 ± 0.02	< 0.0001
HDL-C (mmol/L)	1.38 ± 0.01	1.46 ± 0.01	1.35 ± 0.01	1.31 ± 0.01	< 0.0001
BMI (kg/m^2^)	28.99 ± 0.09	25.46 ± 0.07	29.31 ± 0.08	33.33 ± 0.13	< 0.0001
Physical activity(Mets)	3837.49 ± 65.86	4995.10 ± 105.55	3689.74± 96.11	2465.93± 68.28	< 0.0001
Hypertension, (%)					< 0.0001
No	17799(57.41)	7942(79.43)	5894(60.67)	3963(41.53)	
Yes	13202(42.59)	2389(20.57)	4437(39.33)	6376(58.47)	
Coronary heart disease, (%)					< 0.0001
No	29774(96.04)	10199(98.90)	9971(96.88)	9604(93.30)	
Yes	1227(3.96)	132(1.10)	360(3.12)	735(6.70)	
Stroke, (%)					< 0.0001
No	29819(96.19)	10172(98.86)	9975(97.41)	9672(94.58)	
Yes	1182(3.81)	159(1.14)	356(2.59)	667(5.42)	
Hyperlipidemia, (%)					< 0.0001
No	9579(30.9)	4989(47.59)	2728(25.12)	1862(18.26)	
Yes	21422(69.1)	5342(52.41)	7603(74.88)	8477(81.74)	
T2DM, (%)					< 0.0001
No	25110 (81)	9749(96.03)	8566(87.03)	6795(70.67)	
Yes	5891 (19)	582(3.97)	1765(12.97)	3544(29.33)	

PIR, poverty-to-income ratio; BMI, body mass index; HDL-c, high-density lipoprotein-cholesterol; T2DM, type 2 diabetes mellitus; UA, uric acid; ALT, Alanine aminotransferase; AST, Aspartate aminotransferase.

Continuous variables were presented as mean with standard deviation (mean ± SD), and categorical variables were expressed as proportion.

Continuous variables were analyzed via one-way ANOVA; categorical variables were analyzed using the Chi-square test or the Fisher’s exact test, and P-value less than 0.05 was considered statistically significant.

### 3.2 Association between the WWI and the odds of T2DM

**Figure 2:**
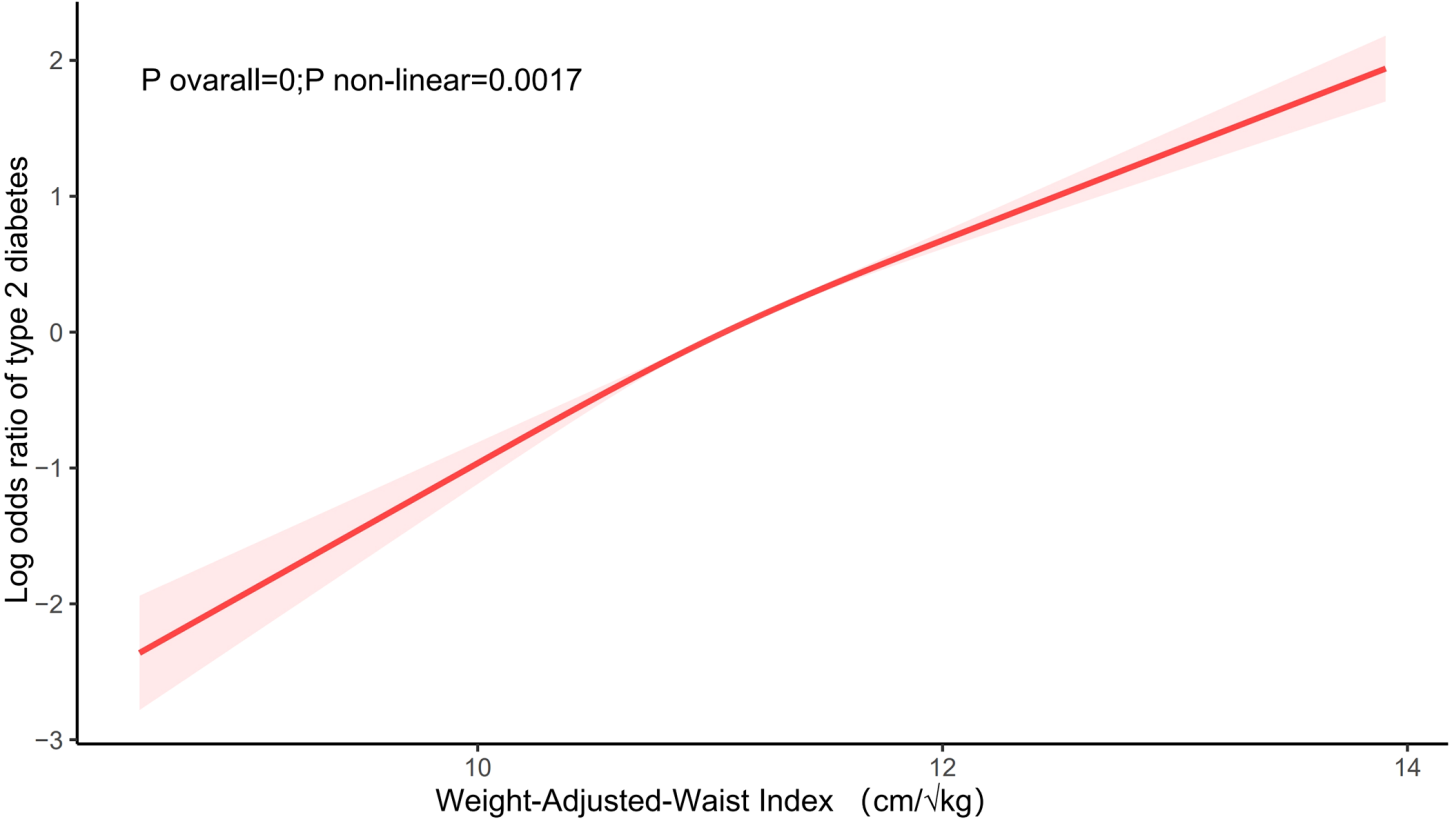
The association between WWI and the odds of T2DM. Age, sex, race/ethnicity, educational level, marital status, PIR, smoking status, alcohol user, Alt, Ast, UA, creatinine, TC, TG, HDL-C, physical activity,hypertension, stroke, coronary heart disease, and hyperlipidemia were adjusted.

As indicated in **Table 2**, in the completely adjusted continuous model, with each 1-unit increase in WWI, the total odds of T2DM increased by 1.14-fold [2.14 [1.98,2.31], P<0.0001]. In the completely adjusted categorical model, compared to the lowest tertile of WWI (T1) serving as the reference group, T2 and T3 were associated with a 0.88-fold [1.88 [1.64,2.17], P<0.0001] and 2.63-fold [3.63 [3.11,4.23], P<0.0001] increase in the odds of developing T2DM, respectively. These findings underscore a significant positive correlation between WWI and the odds of T2DM. Furthermore, the results from the fitted curves indicated a non-linear trend (P non-linear=0.0017), as illustrated in **Figure 2**.

**Table 2:** The association between weight-adjusted-waist index and T2DM.

WWI	Event(%)	Type 2 diabetes OR (95%CI)					
		Model 1	P	Model 2	P	Model 3	P
Per 1 increment	5891(19.00)	3.20(3.00,3.40)	<0.0001	2.73(2.54,2.93)	<0.0001	2.14(1.98,2.31)	<0.0001
Tertiles							
T1	582(3.97)	1.00(reference)		1.00(reference)		1.00(reference)	
T2	1765(12.97)	3.61(3.14, 4.15)	<0.0001	2.59(2.25,2.97)	<0.0001	1.88(1.64,2.17)	<0.0001
T3	3544(29.33)	10.05(8.75,11.53)	<0.0001	6.11(5.29,7.06)	<0.0001	3.63(3.11,4.23)	<0.0001
p for trend			<0.0001		<0.0001		<0.0001

Model 1 was adjusted for none.

Model 2 was adjusted for age, sex, and race/ethnicity.

Model 3 was adjusted for age, sex, race/ethnicity, educational level, marital status, PIR, smoking status, alcohol user, Alt, Ast, UA, creatinine, TC, TG, HDL-C, physical activity,hypertension, stroke, coronary heart disease, and hyperlipidemia.

PIR, poverty-to-income ratio; HDL-c, high-density lipoprotein-cholesterol; UA, uric acid; TG, triglycerides; TC, total cholesterol;ALT, Alanine aminotransferase; AST, Aspartate aminotransferase.

### 3.3 Subgroup analysis

The subgroup analysis was performed to assess the association between WWI and the odds of T2DM in different populations. As shown in **Table 3**, the relationship between WWI and the odds of T2DM was not markedly affected by age, gender, race/ethnicity, hypertension, CHD, stroke, and Hyperlipidemia(P<0.05). Significant interaction (p for interaction < 0.0001) was identified within subgroups based on age(<60、>=60). Among individuals younger than 60 years, each 1-unit increase in WWI was associated with a 1.79-fold elevated odds of T2DM. In contrast, for those aged 60 years or older, each 1-unit increase in WWI was linked with a 0.84-fold increased odds of T2DM. We also found a certain degree of interaction (p for interaction=0.01) in the hypertension (yes/no) subgroup. In hypertensive populations, each 1-unit increasement in WWI was correlated with a 1.06-fold increased odds of T2DM, and in non-hypertensive populations, each 1-unit increasement in WWI was related to a 1.20-fold increased odds of T2DM.

**Table 3:** Subgroup analysis of the association between weight-adjusted-waist index and diabetes.

Subgroup	T2DM [OR (95% CI)]	p	p for interaction
Age(years old)			< 0.0001
>=60	1.84(1.64,2.06)	<0.0001	
<60	2.79(2.54,3.07)	<0.0001	
Sex			0.7
Female	1.99(1.80,2.21)	<0.0001	
Male	2.27(2.02,2.56)	<0.0001	
Race/ethnicity			0.83
Non-Hispanic White	2.26(2.01,2.54)	<0.0001	
Mexican American	1.83(1.56,2.14)	<0.0001	
Non-Hispanic Black	1.97(1.76,2.21)	<0.0001	
Others	2.00(1.77,2.26)	<0.0001	
Hypertension			0.01
Yes	2.06(1.89,2.24)	<0.0001	
No	2.20(1.93,2.50)	<0.0001	
stroke			0.49
Yes	1.96(1.53,2.51)	<0.0001	
No	2.14(1.97,2.33)	<0.0001	
CHD			0.33
Yes	2.67(1.94,3.68)	<0.0001	
No	2.11(1.95,2.29)	<0.0001	
Hyperlipidemia			0.48
Yes	2.13(1.96,2.32)	<0.0001	
No	2.08(1.76,2.46)	<0.0001	

Age, sex, race/ethnicity, educational level, marital status, PIR, smoking status, alcohol user, Alt, Ast, UA, creatinine, TC, TG, HDL-C, physical activity,hypertension, stroke, coronary heart disease, and hyperlipidemia were adjusted.

PIR, poverty-to-income ratio; HDL-C, high-density lipoprotein cholesterol; UA, uric acid; TG, triglycerides; TC, total cholesterol; ALT, Alanine aminotransferase; AST, Aspartate aminotransferase; T2DM, type 2 diabetes mellitus.

## 4 Discussion

In this cross-sectional study involving 31,001 nationally representative participants, we found a strong positive correlation between WWI values and the odds of type 2 diabetes, implying that individuals with a higher WWI are more likely to have type 2 diabetes. Moreover, in the subgroup analyses, we found an interaction between age and hypertension in both subgroups.

A prospective cohort study from Northeast China (n=9205) observed a significant positive correlation between WWI values and the occurrence of type 2 diabetes and suggested that WWI could be a simple and effective predictor for the diagnosis of type 2 diabetes mellitus in a rural population in China (14). Sun H et al. (15) conducted a secondary analysis of a retrospective cohort study in a Japanese population (n=15,464) and found that WWI is a new metabolic index that can be used to predict T2DM in a Japanese population. WWI was found to be a novel metabolic index that can be used to predict T2DM occurrence in the Japanese population. Our findings were generally consistent with the results of these two studies. In addition, subgroup analyses revealed that WWI was more strongly associated with T2DM in people younger than 60 years of age (p for interaction< 0.0001), which may be related to changes in body composition such as significant increases in adiposity and decreases in muscle mass in older adults due to aging (20, 21), and other studies have suggested that it may also be related to differences in adiposity distribution between the younger and the older population (22, 22). differently in younger and older populations (22, 23). Our study also found higher odds of T2DM in the nonhypertensive population than in the hypertensive population.

In addition to being associated with T2DM, WWI is significantly associated with a variety of cardiovascular diseases and poor prognosis. A prospective cohort study from China included 10,338 nonhypertensive subjects with a mean follow-up of 6 years and found that high WWI was significantly linked to an increased risk of hypertension (24).In a cross-sectional study that included 21,040 subjects, Fang H et al. found that high levels of WWI were significantly associated with an elevated risk of development of CVD, especially prominent in those under 50 years of age, suggesting that WWI may be an interventional indicator for reducing the risk of cardiovascular disease in the general adult population (25). In a prospective cohort study (n=26822) with a mean follow-up of 69 months, elevated levels of WWI were found to be independently correlated with an increased risk of cardiovascular mortality and all-cause mortality (26). Zhang D et al. also reported that WWI was associated with heart failure, suggesting that WWI may be a significant marker of heart failure predictability (27). Ye J et al. found in a cross-sectional study of 23,389 cases that larger WWI might be an independently predictive factor for stroke (28). In addition, Cai S et al. have reported that WWI is associated with left heart ventricular fertilization, suggesting that WWI may be an important predictor of cardiometabolic risk (29).

BMI and WC, the traditional measures of obesity, are strongly associated with the development of diabetes mellitus. J M Chan et al. conducted a cross-sectional study that included 51,529 men aged 40-75 years and found that those with a BMI greater than 35 kg/m2 had a substantially increased risk of type 2 diabetes mellitus compared to those with a BMI less than 23 kg/m2 (30). A cohort study from Colombia that included 6,580 subjects with a mean follow-up of 12 years resulted in the definition of waist circumference thresholds of 89 cm in men and 86 cm in women, which were used to identify an elevated risk of developing diabetes (31). A retrospective cohort study from Japan, which included 4754 subjects, found that both BMI and WC were positively associated with the risk of developing diabetes (32). The obesity paradox is still present despite the growing evidence that these traditional obesity markers are correlated with type 2 diabetes mellitus (33). The reason for the controversy may partly stem from the inability of traditional indices to differentiate between fat distribution and muscle mass (34, 35).WWI can accurately reveal centripetal obesity independent of body weight (13). Previous studies have shown that high WWI values are associated with unfavorable body composition outcomes such as high-fat content, low muscle mass, and low bone mass (36). Thus, WWI may serve as a more comprehensible and precise measurement of obesity, reflecting the correlation between obesity and type 2 diabetes mellitus. In recent studies, WWI has been identified as a robust predictor of multiple diseases, superior to BMI and WC (12, 18, 37). WWI is more consistent and reliable in predicting the occurrence of diseases in different races and populations, especially in cross-racial or multicenter studies (37). Regarding other novel indicators such as waist-to-hip ratio (WHR) and waist-to-height ratio (WHtR), which have been used in diabetes research, there is a lack of comparative studies on the comparison with WWI, which may be one of the directions for future studies evaluating obesity and diabetes.

Several underlying mechanisms could account for this positivity in the relationship between WWI and T2DM. Firstly, the increase in WWI may reflect adipose tissue dysfunction, especially the accumulation of visceral adipose tissue, and the large accumulation of visceral fat leads to the release of large quantities of pro-inflammatory factors, including leptin and aldosterone, which lead to insulin resistance and inflammatory responses occurring that can lead to diabetes mellitus (38). Second, adipocytes in centripetally obese individuals are characterized by a hyperlipolytic state that is resistant to the antilipolytic effects of insulin, and the resulting flow of nonesterified fatty acids to the liver not only impairs hepatic metabolism but also leads to an increase in hepatic gluconeogenesis (39). Finally, adipose tissue in obese individuals releases more reactive oxygen species (ROS), and excessive ROS not only reduces the biological availability of nitric oxide (NO) but also hyperoxides tend to react with NO to generate deleterious hydrogen peroxide, which ultimately leads to the development of endothelial cell dysfunction (40, 41). Previous studies have shown that the development of diabetes mellitus is closely related to endothelial dysfunction (42–44).

The advantage of this study is that it is based on NHANES data which were gathered using a graded multi-stage probability sampling strategy, thus making the study more trustworthy and representational. However, our study has several limitations. First, since the current study is a cross-sectional study, causal conclusions cannot be drawn from our results. Second, although we adjusted for various confounders in our model, we could not exclude the effects of other unmeasured potential confounders, such as dietary intake and environmental exposures. Finally, the results may not be extrapolated to other countries because of differences between countries.

## 5 Conclusions

We report that WWI is strongly associated with the odds of T2DM in U.S. adults and found higher odds of T2DM among young adults and non-hypertensive populations. Future studies should focus on the role of different obesity indices in the development of diabetes.

## Data availability statement

The raw data supporting the conclusions of this article will be made available by the authors, without undue reservation.

## Ethics statement

The studies involving humans were approved by National Health and Nutrition Examination Survey center in the United States. The studies were conducted in accordance with the local legislation and institutional requirements. The participants provided their written informed consent to participate in this study.

## Author contributions

DZ: Writing – original draft. SZ: Methodology, Conceptualization, Formal analysis, Validation, Visualization, Writing – review & editing. DL: Data curation, Funding acquisition, Supervision, Writing – review & editing. FL: Supervision, Project administration, Validation, Writing – review & editing. ZR: Methodology, Writing – review & editing. XD: Data curation, Funding acquisition, Writing – review & editing. WC: Funding acquisition, Supervision, Writing – review & editing.
